# miR-155 and miR-484 Are Associated with Time to Progression in Metastatic Renal Cell Carcinoma Treated with Sunitinib

**DOI:** 10.1155/2015/941980

**Published:** 2015-05-03

**Authors:** Jana Merhautova, Renata Hezova, Alexandr Poprach, Alena Kovarikova, Lenka Radova, Marek Svoboda, Rostislav Vyzula, Regina Demlova, Ondrej Slaby

**Affiliations:** ^1^Department of Pharmacology, Faculty of Medicine, Masaryk University, 625 00 Brno, Czech Republic; ^2^Central European Institute of Technology, Masaryk University, 625 00 Brno, Czech Republic; ^3^Department of Comprehensive Cancer Care, Masaryk Memorial Cancer Institute, 656 53 Brno, Czech Republic

## Abstract

*Background*. Sunitinib is a tyrosine kinase inhibitor used in the treatment of metastatic renal cell carcinoma. The main difficulty related to the treatment is the development of drug resistance followed by rapid progression of the disease. We analyzed tumor tissue of sunitinib treated patients in order to find miRNAs associated with therapeutic response. *Methods*. A total of 79 patients with metastatic renal cell carcinoma were included in our study. miRNA profiling in tumor tissue samples was performed by TaqMan Low Density Arrays and a group of selected miRNAs (miR-155, miR-374-5p, miR-324-3p, miR-484, miR-302c, and miR-888) was further validated by qRT-PCR. Normalized data were subjected to ROC and Kaplan-Meier analysis. *Results*. We reported decreased tissue levels of miR-155 and miR-484 as significantly associated with increased time to progression (miR-155: median TTP 5.8 versus 12.8 months, miR-484: median TTP 5.8 versus 8.9 months). *Conclusion*. miR-155 and miR-484 are potentially connected with sunitinib resistance and failure of the therapy. miR-155 is a known oncogene with direct influence on neovascularization. Biological role of miR-484 has to be clarified. Stratification of patients based on miRNA analysis would allow more personalized approach in therapy of metastatic renal cell carcinoma.

## 1. Introduction

Targeted therapy with tyrosine kinase inhibitors (TKIs) is used in the first line of metastatic renal cell carcinoma (mRCC) treatment. TKIs inhibit multiple receptor tyrosine kinases needed for the activation of intracellular signaling pathways controlling cell proliferation, survival, or angiogenesis. Almost all treated patients will eventually develop secondary resistance to TKIs [1]. Other therapeutic alternatives, such as TKIs pazopanib or sorafenib, mTOR inhibitor temsirolimus, VEGFR antibody bevacizumab, cytokine therapy with interferon-*α*, or clinical trials [2], could be provided, if there would be a possibility to distinguish individuals with and without benefit from sunitinib therapy.

Emerging evidence suggests that microRNAs (miRNAs) could be suitable biomarkers with diagnostic, prognostic, and predictive potential [3–6]. These small (18–25 nt) noncoding RNAs are posttranscriptional regulators of gene expression. miRNAs affect most cellular processes and the dysregulation of their network has been linked to various malignant diseases including RCC [7]. miRNAs as biomarkers could be measured in tissues and body fluids and are relatively resistant to decay. The aim of our study was to find tissue miRNAs associated with the time to progression of mRCC in patients treated with sunitinib. To have an effective tool for distinguish patients according to the expected therapy outcome would contribute to more personalized mRCC therapy.

## 2. Materials and Methods

### 2.1. Study Design, Patients, and Tissue Samples

The study protocol was approved by the local ethical committee and written informed consent was obtained from all patients. Metastatic RCC patients included in the study were from South Moravian region of Czech Republic with uniform exposure to the environmental factors. Hereditary cases of RCC were excluded from the study. Two cohorts of patients with mRCC treated with sunitinib in a standard regimen were set up retrospectively. The screening group included 16 patients from Masaryk Memorial Cancer Institute, Brno, Czech Republic (MMCI). Response to the treatment was assessed according to RECIST criteria after 9 months and patients were divided into two groups: (a) responders to the treatment (complete, or partial response, and stable disease) and (b) nonresponders with rapid progression. A group of candidate miRNAs was chosen and the expression was analyzed by qRT-PCR in the validation cohort of 63 mRCC patients from MMCI. Clinicopathological characteristics of both cohorts are summarized in Table 1.

### 2.2. Tissue Samples and RNA Isolation

Tumor tissue was provided as formalin-fixed paraffin embedded (FFPE) samples. Total RNA enriched with small RNA was isolated using mirVana miRNA Isolation Kit (Ambion, Austin, USA). Concentration and purity of the isolated RNA were determined spectrophotometrically using Nanodrop ND-1000 (Thermo Scientific, Rockford, USA).

### 2.3. Microarray Profiling

miRNAs profiling was conducted using TaqMan Low Density Array (TLDA) technology. Megaplex miRNA RT primers set (pools A and B, version 3.0, Applied Biosystems, Foster City, USA) and TaqMan MicroRNA Reverse Transcription kit (Applied Biosystems) were used for reverse transcription. Reactions were carried out according to the manufacturer's protocol. 667 miRNAs were simultaneously quantified using ABI 7900 HT Instrument (Applied Biosystems).

### 2.4. RT-PCR Quantification

Gene-specific primers were used in reverse transcription according to the TaqMan MicroRNA Assay protocol (Applied Biosystems). qRT-PCR was performed on ABI 7500 HT Instrument (Applied Biosystems) using the Applied Biosystems 7500 Sequence Detection System. TaqMan (NoUmpErase UNG) Universal PCR Master Mix and specific primer and probe mix (Applied Biosystems) for each miRNA were used. PCR reactions were run in duplicates, and average threshold cycles and SD values were calculated.

### 2.5. Data Normalization and Statistical Analysis

Expression data from TLDA profiling were normalized using miR-625^∗^, which was uniformly expressed in all samples from screening cohort. Normalized miRNA expression data were evaluated using Bioconductor Limma differential expression analysis. *P* value lower than 0.01 was selected according to the potential of identified miRNAs to accurately discriminate responders and nonresponders in consequent HCL analysis. In validation phase of the study, average expression levels of miRNAs in RT-PCR quantification were normalized using miR-1233 as a reference gene. miR-1233 was selected according to our previous experience with normalization of renal cell carcinoma FFPE samples. Normalized expression data were evaluated by ROC analysis (MedCalc 14.12.0) and Kaplan-Meier analysis followed by log-rank test (GraphPad Prism 5.03). *P* values lower than 0.05 were considered statistically significant.

## 3. Results

### 3.1. Microarray Profiling Revealed 19 Differentially Expressed miRNAs between the Responders and Nonresponders Group

**Figure 1:**
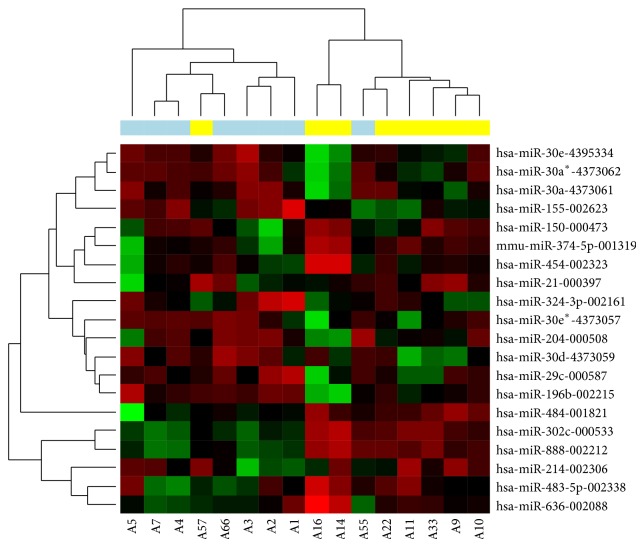
Hierarchical clustergram of miRNAs differentially expressed in sunitinib responding and nonresponding patients. Cluster analysis groups samples and miRNAs according to the expression similarity. miRNAs are in rows and samples in columns. Upregulated miRNAs are marked as red and downregulated miRNAs as green. Blue color indicates responders, yellow color indicates nonresponders. *P* < 0.01.

High-throughput miRNA analysis of tumor tissue of 16 patients treated with sunitinib belonging to either responding (*N* = 8) or nonresponding (*N* = 8) group was performed. Limma analysis of normalized expression data identified 19 miRNAs differentially expressed (Figure 1). Six miRNAs (miR-155, miR-374-5p, miR-324-3p, miR-484, miR-302c, and miR-888) were chosen as candidates for the verification using qRT-PCR (*P* value < 0.01, C_T_ < 35).

### 3.2. Association between miR-155 and miR-484 Expression and Time to Progression in mRCC Treated with Sunitinib

**Figure 2:**
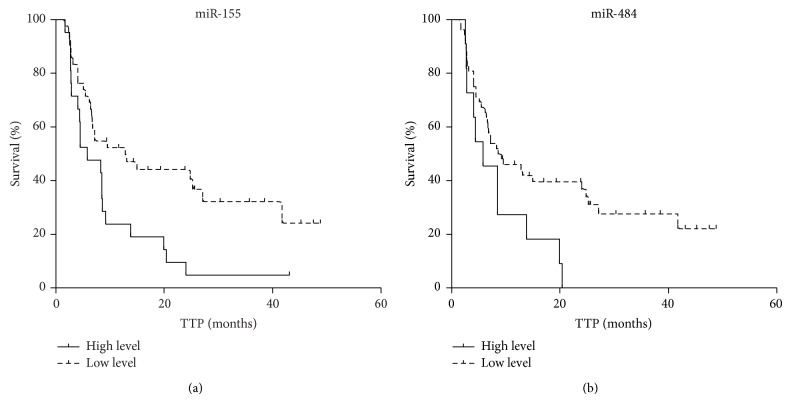
Kaplan-Meier survival curves estimating TTP in sunitinib treated mRCC patients (*N* = 63) according to miR-155 ((a); *P* value < 0.01) and miR-484 ((b); *P* value < 0.05) tumor tissue expression levels. Patients with low expression of the relevant miRNA are illustrated by dashed line.

The results obtained from the screening cohort were verified on the independent cohort (*N* = 63) by qRT-PCR. Normalized data were analyzed by ROC analysis and patients were separated into two groups according to the calculated criterion. Kaplan-Meier analysis revealed that lower level of miR-155 is associated with increased time to progression in patients on sunitinib treatment (Table 2 and Figure 2(a), median TTP 5.8 versus 12.8 months). Similar result was obtained for miR-484 (Table 2 and Figure 2(b), median TTP 5.8 versus 8.9 months). Kaplan-Meier plots of other miRNAs did not reach statistical significance, although some of them indicate potentially interesting trends (data not shown).

## 4. Discussion

Our findings suggest a link between two miRNAs (miR-155 and miR-484) and disease progression in mRCC patients treated with sunitinib. Tyrosine kinase inhibitors inhibit tyrosine kinase domains of growth factor receptors, albeit their main activity is promoted by the inhibition of VEGF receptor cascade, which leads to the decrease in blood tumor perfusion and to the inhibition of neovascularization. Tumors of TKI treatment-refractory patients are able to escape from the VEGFR blockade [1]. miR-155 is a potent oncomiR upregulated in diverse types of cancer including renal cancer [8, 9], which is in accordance with our findings. The role of miR-155 in angiogenesis is well described. Positive feedback loop between VEGF and miR-155 exists, and miR-155 decreases the expression of VHL tumor suppressor, a protein with ubiquitin ligase activity sequestrating, for example, hypoxia-induced factors (HIFs). Higher levels of HIFs promote expression of genes involved in angiogenesis, proliferation, and other aspects of the tumorigenesis, even in the condition of VEGFR blockade [10, 11].

Our data imply that patients with higher tissue expression of miR-155 have decreased time to progression on sunitinib treatment and thus limited benefit from the therapy. However, we have detected a discrepancy between the results obtained from the screening and independent cohort. TLDA screening indicated that the nonresponders from the screening group have lower expression of miR-155 than the responders. Opposite result was achieved by qRT-PCR in the independent cohort (data not shown). We suppose that a bias might occur due to a small number of the specimens analyzed by TLDA, which is also significant limitation of our study.

The expression of miR-484 in mRCC patients treated with sunitinib has already been noticed. Prior et al. described high tumor tissue levels of miR-484 as significantly associated with decreased TTP and overall survival [12]. Our findings are in agreement with this study.

Research in ovarian cancer proved that miR-484 is excreted from tumor cells as a paracrine regulator of tumor microenvironment [13] and it is also measurable in plasma [14, 15]. Therefore, it was found decreased in the tumor tissue [13] and increased in plasma [16]. However, adrenocortical cancer is typical with high tissue expression of miR-484 [17]. The role of this miRNA is probably diverse and depends on the tumor type and miRNA localization. Up to date, there are no reports of possible targets of miR-484 in renal cell carcinoma. Its paracrine function was described in ovarian cancer, where miR-484 targets VEGF B in tumor cells and VEGFR2 in adjacent endothelial cells [13]. Increased levels of miR-484 attenuate the intrinsic apoptotic pathway rising from mitochondria in anoxia, which was unveiled in experiments with myocardial infarction [18].

Independent validation of our results in responders and nonresponders to the sunitinib treatment on larger cohorts of patients and functional analysis of miR-155/miR-484 regulatory involvement in VEGFR signaling might help to understand the underlying mechanism of sunitinib resistance and also prove the potential of these miRNAs to serve as a suitable predictive biomarkers in mRCC patients treated with sunitinib.

## Figures and Tables

**Table 1:** Clinicopathological characteristics of patients.

	Screening cohort		Validation cohort	
	Responders *N* = 8	Nonresponders *N* = 8	Responders *N* = 44	Nonresponders *N* = 19
Gender				
Male	6 (75%)	8 (100%)	34 (77.3%)	11 (57.9%)
Female	2 (15%)	0 (0%)	10 (22.7%)	8 (42.1%)
Age				
Median	64	64	66	66
Range	40–80	53–73	41–84	45–84
Histology				
Papillary carcinoma	1 (12.5%)	1 (12.5%)	3 (6.8%)	3 (5.8%)
Clear cell carcinoma	7 (87.5%)	7 (87.5%)	41 (93.2%)	16 (84.2%)
Grade				
1	0 (0%)	0 (0%)	6 (13.6%)	0 (0%)
2	2 (25%)	3 (37.5%)	11 (25%)	5 (26.4%)
3	5 (62.5%)	3 (37.5%)	13 (29.5%)	7 (36.8%)
4	1 (12.5%)	2 (25%)	5 (11.4%)	7 (36.8%)
Unknown	0 (0%)	0 (0%)	9 (20.5%)	0 (0%)
Response to sunitinib according to RECIST criteria				
Complete response	0 (0%)	0 (0%)	1 (2.3%)	0 (0%)
Partial response	6 (75%)	0 (0%)	19 (43.2%)	0 (0%)
Stable disease	2 (25%)	0 (0%)	24 (54.5%)	0 (0%)
Progressive disease	0 (0%)	8 (100%)	0 (0%)	19 (100%)

**Table 2:** Validation of miR-155 and miR-484 on the independent cohort (*N* = 63) and their correlation with TTP (months).

	Number of patients (*N* = 63)	Median TTP (months)	Log-rank *P*	HR	95% CI
miR-155					
Low, <0.2381	42	12.8	0.0092	2.412	1.243–4.680
High, ≥0.2381	21	5.8			
miR-484					
Low, <1.4408	52	8.9	0.0296	2.623	1.100–6.254
High, ≥1.4408	11	5.8			

## References

[1] Rini B. I., Atkins M. B. (2009). Resistance to targeted therapy in renal-cell carcinoma. *The Lancet Oncology*.

[2] Bex A., Kroon B. K., de Bruijn R. (2012). Is there a role for neoadjuvant targeted therapy to downsize primary tumors for organ sparing strategies in renal cell carcinoma?. *International Journal of Surgical Oncology*.

[3] Mlcochova H., Hezova R., Stanik M., Slaby O. (2014). Urine microRNAs as potential noninvasive biomarkers in urologic cancers. *Urologic Oncology: Seminars and Original Investigations*.

[4] Wang C., Hu J., Lu M. (2015). A panel of five serum miRNAs as a potential diagnostic tool for early-stage renal cell carcinoma. *Scientific Reports*.

[5] Mishra P. J. (2014). MicroRNAs as promising biomarkers in cancer diagnostics. *Biomarker Research*.

[6] Hansen T. F., Carlsen A. L., Heegaard N. H., Sørensen F. B., Jakobsen A. (2015). Changes in circulating microRNA-126 during treatment with chemotherapy and bevacizumab predicts treatment response in patients with metastatic colorectal cancer. *British Journal of Cancer*.

[7] White N. M. A., Bao T. T., Grigull J. (2011). MiRNA profiling for clear cell renal cell carcinoma: biomarker discovery and identification of potential controls and consequences of miRNA dysregulation. *The Journal of Urology*.

[8] Li S., Chen T., Zhong Z., Wang Y., Li Y., Zhao X. (2012). MicroRNA-155 silencing inhibits proliferation and migration and induces apoptosis by upregulating BACH1 in renal cancer cells. *Molecular Medicine Reports*.

[9] Wojcicka A., Piekielko-Witkowska A., Kedzierska H. (2014). Epigenetic regulation of thyroid hormone receptor beta in renal cancer. *PLoS ONE*.

[10] Biswas S., Troy H., Leek R. (2010). Effects of HIF-1*α* and HIF2*α* on growth and metabolism of clear-cell renal cell carcinoma 786-0 xenografts. *Journal of Oncology*.

[11] Kong W., He L., Richards E. J. (2014). Upregulation of miRNA-155 promotes tumour angiogenesis by targeting VHL and is associated with poor prognosis and triple-negative breast cancer. *Oncogene*.

[12] Prior C., Perez-Gracia J. L., Garcia-Donas J. (2014). Identification of tissue microRNAs predictive of sunitinib activity in patients with metastatic renal cell carcinoma. *PLoS ONE*.

[13] Vecchione A., Belletti B., Lovat F. (2013). A microRNA signature defines chemoresistance in ovarian cancer through modulation of angiogenesis. *Proceedings of the National Academy of Sciences of the United States of America*.

[14] Li A., Yu J., Kim H. (2013). MicroRNA array analysis finds elevated serum miR-1290 accurately distinguishes patients with low-stage pancreatic cancer from healthy and disease controls. *Clinical Cancer Research*.

[15] Zearo S., Kim E., Zhu Y. (2014). MicroRNA-484 is more highly expressed in serum of early breast cancer patients compared to healthy volunteers. *BMC Cancer*.

[16] Kjersem J. B., Ikdahl T., Lingjaerde O. C., Guren T., Tveit K. M., Kure E. H. (2014). Plasma microRNAs predicting clinical outcome in metastatic colorectal cancer patients receiving first-line oxaliplatin-based treatment. *Molecular Oncology*.

[17] Bimpaki E. I., Iliopoulos D., Moraitis A., Stratakis C. A. (2010). MicroRNA signature in massive macronodular adrenocortical disease and implications for adrenocortical tumourigenesis. *Clinical Endocrinology*.

[18] Wang K., Long B., Jiao J.-Q. (2012). MiR-484 regulates mitochondrial network through targeting Fis1. *Nature Communications*.

